# Predictive Factors for Pressure Ulcers in an Older Adult Population Hospitalized for Hip Fractures: A Prognostic Cohort Study

**DOI:** 10.1371/journal.pone.0169909

**Published:** 2017-01-09

**Authors:** Paolo Chiari, Cristiana Forni, Monica Guberti, Domenica Gazineo, Sabrina Ronzoni, Fabio D’Alessandro

**Affiliations:** 1 Dipartimento Scienze Mediche e Chirurgiche, Università di Bologna, Bologna, Italy; 2 Centro ricerca professioni sanitarie, Istituto Ortopedico Rizzoli, Bologna, Italy; 3 Ricerca & EBP, Arcispedale Santa Maria Nuova-IRCCS, Reggio Emilia, Italy; 4 Centro Studi EBN, Azienda Ospedaliero-Universitaria di Bologna, Bologna, Italy; Case Western Reserve University, UNITED STATES

## Abstract

**Background:**

Older adult patients with fragility hip fractures constitute a population at high risk for complications, in particular pressure ulcers. The aim was to evaluate the incidence of pressure ulcers and potential predictive factors.

**Methods and Findings:**

A prospective multicentric prognostic cohort study in orthopedic wards in three Italian public hospitals. Participants were all consecutive patients 65 years of age or older diagnosed with a fragility hip fracture. Outcomes were incidence of pressure ulcers. The exposure variables were grouped into three macro areas in order to facilitate reading: “intrinsic” variables, “extrinsic” variables and variables linked to the organization of patient care. One thousand eighty-three older adult patients with fragility hip fractures were enrolled from October 1^st^, 2013 to January 31^st^, 2015, and pressure ulcers developed in 22.7%. At multivariate analysis, the following were found to be risk factors: age> 80 years (odds ratio (OR) 1.03; p = 0.015), the length of time a urinary catheter was used (OR 1.013; p<0.001), the length of time pain was present (OR 1.008; p = 0.008), the absence of side rails on the bed (OR 1.668; p = 0.026) and the use of a foam position valve (OR 1.025; p<0.001). Instead, the protective factors were the presence of a caregiver for at least half a day daily (OR 0.994; p = 0.012) and the number of positionings during the postoperative period (OR 0.897; p = 0.008).

**Conclusions:**

The study allowed the identification of the patients most at risk for developing pressure ulcers, and the construction of a pragmatic predictive model using significant risk or protective factors in order to reduce the number of pressure ulcers.

## Introduction

In older adults, hip fractures are a serious health problem due to the mortality and disability that they often cause. In addition, they have an important economic impact, both on the health system and on the families involved [1]. Given the increase in the older adult population, especially in Italy, the incidence of these fragility fractures is constantly increasing with 88,647 new cases per year in Italy [2] and 1.5 million all over the world every year [3,4]. Hip fractures are associated with mortality, disability and a decrease in the quality of life [5]. Pressure ulcers constitute the complication most correlated to patient care, having an incidence which varies from 8.8 to 55% in the literature [6,7]. Some studies in the literature have researched the prevalence and incidence of pressure ulcers in patients with fragility hip fractures, attempting to also identify specific risk factors [6,8–13]. However, many of these studies were retrospective [6–10] with contrasting results and were carried out using patient records created for other aims [5]. Furthermore, there were very few prospective studies which investigated potential risk factors, such as pain management [14,15], the different modalities of preoperative immobilization of the fractured limb, and the different nursing and rehabilitative activities of patient care correlated with the management of these patients [16]. The organizational and socio-cultural context regarding patient origin could create situations of risk as has been reported in a European study in which, however, the Italian sample was very small [9].

The aim of the present study was to evaluate the incidence of pressure ulcers from the moment of admittance of older adult patients with a fragile proximal hip fracture to their discharge. Moreover, the study sought to evaluate potential predictive factors linked to medical, nursing and rehabilitative patient care, and to their organization with the aim of identifying the population most at risk and the most efficacious patient care for this population.

## Materials and Methods

A prospective multicentric prognostic cohort study was carried out in three Italian public hospitals. In the hospitals involved in the study, there were various departments which treated patients with fragility fractures using different modalities, regarding both surgical treatment, and nursing and rehabilitative patient care.

The study was approved by the Ethics Committee of Rizzoli Orthopedic Institute (0012688; 13/03/2013) (coordinator of the study), the Ethics Committee of the University Hospital of Bologna (189/2013/O/Oss; 29/10/2013) and by the Ethics Committee of the Hospital of Reggio Emilia (collaborating centers). Written consent was obtained from all patients.

All consecutive patients ≥ 65 years of age admitted to the Emergency Rooms of the participating hospitals with a diagnosis of fragility hip fracture (pertrochanteric, femoral neck and subtrochanteric) were enrolled. Patients with periprosthetic or pathological fractures, and patients who presented with pressure ulcers were excluded. Patients with periprosthetic fractures were, in fact, treated in different ways and with different approaches, therefore having very different care needs. Moreover, they accounted for no more than 2.5% of all fragility fractures.

### Outcomes

Primary: incidence of pressure ulcers [17]. The skin of the patient (occiptal area, scapula, iliac and trochanter crests, sacrum, elbows, heels, back, calves, thighs and ankles) was observed daily.

Secondary: potential predictive factors.

### Predictive factors

All the variables studied in the articles published in the literature were identified and were integrated with those identified by a panel of experts set up for the occasion. The variables were grouped into three macro areas in order to facilitate reading: “intrinsic” variables, “extrinsic” variables and variables linked to the organization of patient care (organizational variables) [5,9].

#### Intrinsic variables

The intrinsic values were: age (in years), gender, prefracture activites of daily living (ADL) [18], residence of the patient before the fracture (resident in their own home or at a social care facility), number and location of other eventual fractures, the presence of comorbidities (Charlson Index) [19], level of risk of developing pressure ulcers (Braden scale) [20], nutrition and evaluation of physical condition divided into three classes (thin, normal, morbid obese), level of dehydration (clinical evaluation at presentation), disorientation during the hospital stay (clinical evaluation), type of fracture and pharmacological therapy at home.

#### Extrinsic and patient care variables

The extrinsic and patient care variables were: thickness of the mattress on the gurney in the Emergency Room (ER), level of pain at admittance to the ER and during hospitalization and rehabilitation, onset of hyperpyrexia, hemoglobin values during hospitalization, typology and length of surgery, length of time in the intensive care unit or in other medical departments in order to stabilize general conditions, restraint (and type), treatment to prevent pressure ulcers (typology of the devices used, such as air mattresses with alternating or static pressure, heel drains, frequency of mobilization carried out by both the patient care and the rehabilitative personnel), management of eventual incontinence (use of diapers, urinary catheter), use of devices for controlling the position of the operated fractured limb in order to contain external rotations (foam valve, sandbags or sheet on the side of the limb, free limb, the use or not of preoperative traction and its typology), the presence of a caregiver during hospitalization, the number of physical therapy sessions actually carried out and the time required with respect to achieving the positions of the patient, assisted or independently (seated, static, ambulatory for the first time).

#### Organizational variables

The organizational variables were: time from the occurrence of the fracture to arrival in the ER, transfer from another hospital, time in the ER, time from arrival in the ER to surgery, and timing with respect to starting physiotherapy.

All the variables were collected until patient discharge or until the appearance of a pressure ulcer. Data regarding the categorical type predictors, where variable during the hospital stay, were collected daily; the percentage of days present/absent of the predictor were also calculated in relation to the days of hospitalization.

### Statistical Analysis

The data obtained were calculated using SPSS v.19.0 (IBM Corp., Armonk, NY, USA). All continuous data were expressed in terms of the mean and the standard deviation of the mean, or median and range when not normally distributed; the categorical data were expressed as frequencies and percentages. The Kolmogorov-Smirnov test was carried out to assess the normality of the continuous variables. The Levene test was carried out to assess homoscedasticity.

Analysis of variance (ANOVA) was carried out to assess the between-group differences of continuous, normally distributed and homoscedastic data; the Mann-Whitney test was used for all other assessments.

Analysis of variance, followed by the Scheffè post hoc pairwise comparison, was also used to assess the between-group differences of the continuous, normally distributed and homoscedastic data; the Kruskal-Wallis test followed by the Mann-Whitney test with the Bonferroni correction for multiple comparisons was used for all other assessments. The Fisher exact test was carried out to investigate the relationships between the dichotomous variables. The Pearson Chi-square test, evaluated using Exact Methods for small samples, was carried out to investigate the relationships between group variables. Logistic regression using the Wald backward method was utilized to find which of the intrinsic factors independently influenced the outcome. This was repeated in order to select which patient care and organizational factors were significant in the univariate analysis and, corrected by the intrinsic factors found with the previous analysis, influenced the outcome.

Receiver operator characteristic (ROC) curve analysis was used to check the logistic regression model.

P<0.05 was considered significant for all tests.

In this study, the number of patients to be enrolled was determined on the basis of the number of predictive parameters inserted into the multivariate analysis. The general empiric rule applied [21] established that the relationship between the total number of events and the number of groups of explicative variables had to be at least ten for each type of variable. On the basis of these considerations, it was estimated to include at least 800 patients.

## Results

**Fig 1:**
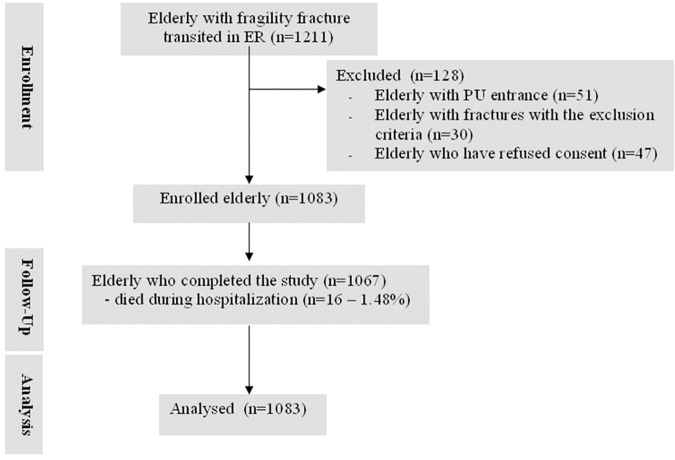
Flow Diagram of older adults.

The study began October 1^st^, 2013 and enrollment ended January 31^st^, 2015. In this period, 1211 patients presented at the ER with a fragility hip fracture of whom 1130 (93.3%) met the inclusion crtieria of the study. Of these, 1083 (95.8%) agreed to participate in the study and were enrolled (Fig 1).

Patient characteristics are reported in Table 1.

**Table 1:** Principal Characteristics and Variables of the Patients.

Variables	Patients without pressure ulcers (n.837)	Patients with pressure ulcers (n.246)	All patients (n. 1083)	p*
**Intrinsic variables:**				
• Average age	83.7 (7.8)	85.6 (6.9)	84.1 (7.6)	0.001
• Female gender	75.4%	74.4%	75.2%	Ns
• Patients without pain at arrival in the ER (NRS or PAINAD score)	37.4%	34.6%	36.8%	Ns
• Average pain at arrival in the ER (NRS or PAINAD score)	4.8 (2.5)	5.0 (2.5)	4.8 (2.5)	Ns
• Femoral neck fracture (vs. trochanteric)	50.3%	56.6%	51.7%	Ns
• Other fractures at presentation	6.6%	9.8%	7.3%	Ns
• Average prefracture Activity Daily Living (ADL)	1.5 (2.0)	1.8 (2.1)	1.6 (2.1)	Ns
• Average risk of developing pressure ulcers (Braden score)	15.7 (2.2)	14.9 (2.4)	15.5 (2.2)	<0.0005
• Average comorbidity (Charlson score)	1.9 (1.8)	2.0 (1.7)	2.0 (1.8)	Ns
• Long-term cortisone therapy	12.8%	12.8%	12.8%	Ns
• Constitutionally very thin (vs. normal or obese)	17.1%	25.5%	19.0%	0.004
• Patients coming from nursing homes (vs. home)	8.6%	9.8%	8.9%	Ns
• Patients never disoriented during hospitalization	41.2%	35.8%	40.0%	Ns
• Average percentage of days of disorientation	22.3 (36.0)	29.1 (40.7)	23.9 (37.2)	0.012
• Average Hblevel at presentation	12.3 (1.7)	12.2 (1.7)	12.3 (1.7)	Ns
**Extrinsic or patient care variables:**				
• Patients positioned on a gurney in the ER having a mattress <6cm	28.1%	24.7%	27.3%	Ns
• Osteosynthesis surgery with an intramedullary nail (vs. arthro- or endoprothesis)	54.2%	60.0%	55.5%	Ns
• Average length of surgery (minutes)	59 (26)	58 (25)	59 (25)	Ns
• Postoperative hospitalization in the intensive care unit	2.7%	5.7%	3.4%	0.025
• PhT	92.6%	74.0%	88.4%	<0.0005
• Average daily preoperative positioning	2.3 (2.2)	1.9 (2.0)	2.2 (2.2)	0.022
• Average daily postoperative positioning	5.6 (2.2)	5.0 (2.5)	5.5 (2.3)	0.001
• Average number of days until drain removal	1.26 (0.68)	1.36 (0.59)	1.28 (0.66)	Ns
• Patients never without diapers	20.8%	26.8%	22.2%	0.045
• Average percentage of days with a diaper	37.8 (36.4)	37.5 (35.4)	37.7 (36.2)	Ns
• Patients without a urinary catheter	3.6%	2.8%	3.4%	Ns
• Average percentage of days with a urinary catheter	53.3 (38.7)	71.6 (36.1)	57.4 (38.9)	<0.0005
• No use of restraints	15.8%	25.2%	17.9%	0.001
• Average percentage of days with restraints (bed rails)	67.4 (37.5)	62.3 (41.3)	66.3 (38.4)	Ns
• Fractured limb without immobilization	23.4%	21.1%	22.9%	Ns
• Average percentage of days with a foam valve under the operated limb	15.3 (21.7)	34.6 (36.0)	19.7 (26.9)	<0.0005
• Patients without an anti-decubitus mattress	30.2%	33.3%	30.9%	Ns
• Foam anti-decubitus mattress	8.8%	11.4%	9.4	Ns
• Anti-decubitus mattress with a motor	60.3%	55.7%	59.3%	Ns
• Patients without heel drains	38.6%	38.6%	38.6%	Ns
• Average percentage of days with a heel drain	74.7 (35.4)	67.8 (39.6)	73.1 (36.5)	0.009
• Average percentage of a decrease in Hb with respect to the initial values	24.2 (11.4)	20.0 (12.1)	23.2 (11.7)	<0.0005
• Incidence of fever	74.8%	63.4%	72.2%	<0.0005
• Patients without pain during hospitalization	21.3%	30.9%	23.5%	0,002
• Average percentage of days with pain ≥ 4 (NRS)	27.5 (26.3)	35.4 (35.2)	29.3 (28.8)	<0.0005
**Organizational variables:**				
• Average number of hours from the occurrence of the fracture to arrival in the ER	21 (56)	29 (136)	23 (82)	0.027
• Hospitalization in orthogeriatrics	43.0%	10.7%	53.7%	0.018
• Transfer to another hospital before arriving at our ER	34.1%	42.7%	36.0%	0.013
• Average wait time in the ER (hours)	1:35 (1:20)	1:30 (0:50)	1:35 (1:15)	Ns
• Average wait time from arrival in the ER to surgery (hours)	39 (29)	42 (27)	40 (28)	Ns
• Operated on within 24 hours	28.4%	27.5%	28.2%	Ns
• Operated on within 48 hours	75.3%	72,1%	74,6%	Ns
• Patients without a caregiver	11.8%	7.3%	10.8%	0.045
• Average percentage of days with a partial caregiver	55.6 (41.7)	58.3 (42.3)	56.2 (41.8)	Ns
• Average number of days from surgery to the start of PhT	1.5 (1.4)	1.8 (1.6)	1.6 (1.4)	0.035

Description of the principal characteristics and variables of the patients with respect to the development of pressure ulcers. The data are reported as median (± SD) or percentages. Missing data: preoperative positionings: 132; days of beginning physiotherapy treatment (PhT): 118; pain at arrival in the Emergency Room (ER): 85; daily postoperative positioning: 72; hours of wait time from the occurrence of the fracture to the arrival in the ER: 63; day of drain removal: 59; Hemoglobin (Hb) values at presentation and perentage of Hb loss: 57; risk of developing pressure ulcers (Braden score): 49; hours of wait time from arrival in the ER to surgery: 37; hours of wait time in the ER: 35; length of surgery: 31; type of surgery (osteosynthesis vs. arthro- or endoprothesis):28; prefracture pressure ulcers: 18; guerney mattress in the ER < 6cm: 13; constitution: 12; cortisone therapy: 4; days with pain: 2.

*The p value is dervied from the comparison of the patients with and without pressure ulcers with respect to that variable, utilizing Fisher’s exact test, the Pearson Chi-square test, the t test or the Mann-Whitney test where appropriate.

NS: Not Significant.

Sixteen older adult patients died while hospitalized (1.48%). The average length of hospitalization was 10.9 days (SD 5,2). The incidence of pressure sores was 22.7% (246 cases) and, on average, they arose on the 5th day of hospitalization (SD 3.7); in 16.2% of the cases, there were multiple locations (40 cases). Altogether, there were 277 pressure ulcers of which 63.9% (177 cases) were located at the sacrum, 22.7% at the heel (63 cases) and 13.4% in other areas (37 cases). The incidence of pressure ulcers ≥ grade 2 was 11.4% (123 cases) S1 Dataset. Univariate analysis revealed that different variables significantly predicted the development of pressure ulcers (Table 1). In order to avoid the presence of confounding factors or multicollinearity, the predictive intrinsic values were studied using multivariate analyses in order to identify those which were independently asociated with the outcome. In the multivariate model, only age ≥81 years and being constitutionally thin were predictors versus being constitutionally normal or obese which were then used as correction factors in successive analyses.

A sucessive multivariate analysis considered the following patient care or organizational variables which were significant in the unvariate analysis: the presence of other fractures at presentation, femoral neck fractures (vs. trochanteric), osteosynthesis surgery with an intramedullary nail (vs. arthro- or endoprosthesis), the number of days until drain removal, disorientation during hospitalization, days with a caregiver for at least one meal, the presence of a heel drain and the number of days of correct restraint for those > 80 years of age who are constitutionally thin vs. those who are constitutionally normal or an obese. The results are reported in Table 2.

**Table 2:** Results of the Multivariate Analyses.

Predictive factor	OR	95% CI	P
Average age	**1.030**	**1.006; 1.054**	**0.015**
Presence of a diaper	**1.555**	**0.980; 2.467**	0.061*
Absence of railings on the bed (restraint)	**1.668**	**1.062; 2.622**	**0.026**
Average daily postoperative positioning	**0.897**	**0.828; 0.971**	**0.008**
Average percentage of days with a urinary catheter	**1.013**	**1.008; 1.018**	**<0.0005**
Average percentage of days with the partial presence of a caregiver	**0.994**	**0.990; 0.999**	**0.012**
Average percentage of days with a foam valve	**1.025**	**1.018; 1.032**	**<0.0005**
Average percentage of days with pain	**1.008**	**1.002; 1.014**	**0.008**

Results of the multivariate analyses carried out for the identification of the predictive factors for pressure ulcers in patients with hip fractures.

*The presence of a diaper with a value close to significance (p = 0.061) improves the predictiveness of the overall model when the other factors remain constant.

The number of rehabilitation sessions and the attainment of a sitting position on the part of the patient were not included as they were influenced to a great extent by the early onset of pressure ulcers which resulted in the patient leaving the study and which involved not collecting the data of the successive rehabilitation sessions.

**Fig 2:**
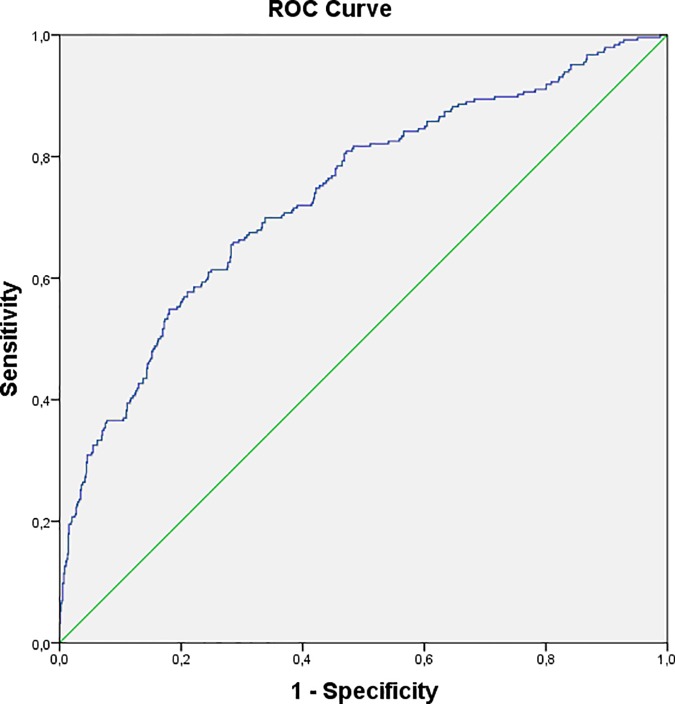
ROC Curve of the Predictive Model of Pressure Ulcers in Patients with Hip Fractures.

The ROC curve (Fig 2) constructed using the probability of verifying the outcome derived from the model resulting from the multivariate analysis had an area under the curve (AUC) of 0.736 (95% confidence interval (CI): 0.699; 0.773).

### Analysis of the Subgroup Having an Operated Fractured Limb without Immobilization

Of the patient care variables, the use of a foam valve was one of the variables which principally predisposed the patient to the risk of pressure ulcers; its influence on the multivariate model was so great that it overshadowed the other predictors.

In order to verify whether other eventual risks or protective factors could have influenced the outcome, an analysis was carried out regarding the subgroup of older adult patients who had never used this system for immobilization of the operated fractured limb. The subgroup included 516 older adult patients. In this subgroup, the incidence of pressure ulcers was 18.2% (94 patients) with respect to 26.8% of the patients who used the valve for even only one day.

Univariate analysis revealed that the use of an alternate pressure mattress was significant with respect to a static anti-decubitus mattress as well as to a standard mattress as reported in Table 3.

**Table 3:** Incidence of pressure ulcers and devices.

	Normal Mattress % (n.)	Foam Mattress % (n.)	Alternating pressure Mattress % (n.)	P
Incidence of pressure ulcers (any grade)	25.6 (23/90)	28.0 (7/25)	16.0 (64/401)	0.044

Use of an anti-decubitus mattress and the development of pressure ulcers in patients without positioning devices on the limb (516 patients).

The result of the new model, built with patients not using a valve, is described in Table 4.

**Table 4:** Results of the multivariate analyses in patients without immobilization of the operated fractured limb.

Predictive factor	OR	95% CI	P
Age	**1.068**	**1.024; 1.114**	**0.002**
Average daily postoperative positioning	**0.689**	**0.598; 0.795**	**<0.001**
Days of wait time until beginning physiotherapy	**1.182**	**1.038; 1.346**	**0.012**
Absence of an anti-decubitus mattress with a motor	**3.715**	**1.061; 13.007**	**0.040**

Results of the multivariate analyses carried out for the identification of the predictive factors of pressure ulcers in the subgroup of patients without immobilization of the operated fractured limb.

The model resulting from the multivariate analysis of the subgroup without mobilization of the operated fractured limb had a ROC curve with an AUC of 0.754 (95% CI: 0.694; 0.815).

## Discussion

The average incidence of pressure ulcers was 22.7% while, for those of grade 2 or higher, it was 11.4%. This incidence was the same as the average incidence described in the literature [6,9,11–13].

Of all the intrinsic characteristics of the patients involved, only age > 80 years was found to be significant. Using all the other patient care and organizational variables in the multivariate analysis, being constitutionally thin was not predictive. This result agreed with the majority of the studies published [6,8–12]. Contrarily to what has been pointed out in some studies [6,10], the prefracture abilities in the present study were not predictive factors which was in accordance with other studies [9,11]. This can be ascribed to the rehabilitative modalities (timing and intensity) which the hospitals participating in the study applied rapidly and independently of prefracture abilities. The presence of comorbidities and the hydration conditions were also found not to be predictors, a fact which contrasted with the results reported in the literature. The studies which indicated them as such had designs at a greater risk of bias [10,15] or involved populations with different characteristics [14]. Living at home before the fracture rather than in an institution was also found not to be a predictor, and neither were gender nor cortisone therapy.

As regards patient care and organizational variables, it was observed that the patient care maneuver involving the most risk was the use of a foam valve (a variable not evaluated in other studies) and not using anti-decubitus mattresses with a motor with respect to static ones. Mattresses with a motor are preventive instruments which have already been studied in the literature [22]; however, the use of a valve nullifies the preventive efficacy. The use of a valve to fix the fractured limb and then operated in a container which maintains the correct position preventing paralysis of the external popliteal sciatic nerve is very frequent in Italian orthopedics, even if it is not universally applied. However, this complication is of marginal incidence (6 cases) and equally distributed between the patients who used the valve and those who did not. Frequent positioning also had a protective effect (the greater the number of positioning the lower the number of pressure ulcers) confirming what was recommended in the guidelines [22] and in contrast to what was identified in the European study [9]. Surprisingly, anemia (with respect to the entry value) was a protective factor at univariate analysis, but this predictor disappeared after correction for the age of the sample. In fact, for younger patients, more invasive surgery is preferred (arthroprothesis); this causes greater loss of blood but guarantees better functionality for a longer period of time. The studies which considered this factor had contrasting results [6,9,13, 23]. Interestingly, the absence of pain was predictive of pressure ulcers at univariate analysis but this unexplainable relationship disappeared in the multivariate model, probably since it was a confounding variable. The use of railings on the bed, as a system of restraint, was found to be efficacious in decreasing pressure ulcers, probably due to the fact that they allowed some movement on the part of the patient. The only study which evaluated restraint systems did not identify the predictors of lesions and did not specify which systems were being evaluated, in particular the retrospective study of Baumgarten [15]. Possible risk factors were the continuous use of a urinary catheter and the use of a diaper while the presence of a caregiver was a protective factor. Even in the latter cases, there are no studies which analyzed these variables but, with respect to the use of urinary catheters in older adult patients, there have been recommendations for their early postoperative removal [24] in order to prevent urinary infections. Furthermore, an important protective factor was the early start of postoperative rehabilitation which confirmed the importance of mobilization in these patients. Instead, the thickness of the mattress on the gurney in the ER was confirmed not to influence the development of lesions [9]. It should be noted that the time in the ER, and therefore on the gurney, was only 1 hour and 35 minutes, much less than that indicated in other studies [25]. Other results which contrasted with those reported in the literature regarded the length of time to surgery; this did not have any correlation with the development of pressure ulcers as has instead been indicated by the majority of studies [6,10,25]. However, the average wait (40 hours) was less than that stated in the above-mentioned studies which confirmed the value of early surgery. Transfer to the intensive care unit and the length of surgery were not identified as possible predictors, unlike what has been observed in other studies [6,10,12]; however, possible confounders (type of patient care furnished in the intensive care unit, anti-decubitus devices used in the operating room) make comparison with the literature difficult. Another patient care variable not correlated with the development of pressure ulcers is osteosynthesis surgery with respect to an endo- or arthroprothesis; this result is in line with what has been found in other studies which have evaluated this variable [10,12]. The model analyzed using the ROC curve showed good predictability for pressure ulcers (AUC: 70–77%). This result was better than that for other instruments specifically utilized for evaluating acute geriatric events (AGEs)[26]. The model was also good in the subgroup analyzed without a valve (AUC: 69–82%).

The principal limitation of the present study was the lack of some information which probably weakened the power of the multivariate analyses; the results did not substantially modify the model. Another limitation was the fact that the constitution of the patients was not evaluated using the body mass index (BMI) but with a clinical evaluation; this reduced the precision of the data but allowed us to be more pragmatic in that clinical evaluation is used more frequently than objective measurement. In our opinion, this aspect favors the generalizability of the results and their ability to be transferred to various practical contexts; the same considerations also applied to the disorientation variable. Instead, for the evaluation of dementia, an objective instrument was used as effectively happens in clinical practice.

## Conclusions

The present study demonstrated that the patients most at risk of developing pressure ulcers were the older adults > 80 years of age, regardless of other intrinsic characteristics, and that the most protective patient care practices were frequent postoperative positioning, the early start of rehabilitation, positioning railings on the bed, the use of anti-decubitus mattresses with a motor, early removal of eventual urinary catheters and limiting the use of diapers to manage fecal incontinence. Moreover, it is necessary to avoid the use of valves to maintain the operated limb in position, and to promote, where possible, the presence of a caregiver for at least a few hours a day.

## Supporting Information

S1 STROBE Statement Checklist of items that should be included in reports of observational studies. (DOC)

S1 Dataset Data underlying the findings. (XLS)
