# Supplementary material for: Predictive Factors for Pressure Ulcers in an Older Adult Population Hospitalized for Hip Fractures: A Prognostic Cohort Study
Source: PLoS One. 2017 Jan 9;12(1):e0169909. doi: 10.1371/journal.pone.0169909 (PMC5222344; doi:10.1371/journal.pone.0169909)
Supplement: S1 STROBE Statement — (DOC) [file pone.0169909.s001.doc]

STROBE Statement—checklist of items that should be included in reports of observational studies

Bologna 2016-05-17, Paolo Chiari, corresponding author

|  | | Item No | Recommendation | Author’s comment and page referral |  |
| --- | --- | --- | --- | --- | --- |
| **Title and abstract** | | 1 | (*a*) Indicate the study’s design with a commonly used term in the title or the abstract | Stated in title, title page  Abstract; first paragraph under Methods and Findings |  |
|  |  |  | (*b*) Provide in the abstract an informative and balanced summary of what was done and what was found | Abstract; paragraphs under Background |  |
| Introduction | | | |  |  |
| Background/rationale | | 2 | Explain the scientific background and rationale for the investigation being reported | Introduction; first and second paragraphs; page 2-3 |  |
| Objectives | | 3 | State specific objectives, including any prespecified hypotheses | Introduction; third paragraph; page 3 |  |
| Methods | | | |  |  |
| Study design | | 4 | Present key elements of study design early in the paper | Methods; second paragraph; page 3 |  |
| Setting | | 5 | Describe the setting, locations, and relevant dates, including periods of recruitment, exposure, follow-up, and data collection | Methods; second paragraph; page 3  Results; first paragraph; page 6 |  |
| Participants | | 6 | (*a*) *Cohort study*—Give the eligibility criteria, and the sources and methods of selection of participants. Describe methods of follow-up | Methods; third paragraph; page 3 |  |
|  |  |  | (*b*) *Cohort study*—For matched studies, give matching criteria and number of exposed and unexposed | N/A |  |
| Variables | | 7 | Clearly define all outcomes, exposures, predictors, potential confounders, and effect modifiers. Give diagnostic criteria, if applicable | Methods/Outcomes; first and second paragraphs; page 4.  Methods/Predictive factors; all paragraphs; page 4-5. |  |
| Data sources/ measurement | | 8* | For each variable of interest, give sources of data and details of methods of assessment (measurement). Describe comparability of assessment methods if there is more than one group | Methods/Predictive factors; the last paragraph; page 5. |  |
| Bias | | 9 | Describe any efforts to address potential sources of bias | Methods/Predictive factors; the first paragraph - Page 4 |  |
| Study size | | 10 | Explain how the study size was arrived at | Methods/Statistical Analysis; the last paragraph; page 6. |  |
| Quantitative variables | | 11 | Explain how quantitative variables were handled in the analyses. If applicable, describe which groupings were chosen and why | Methods/Predictive factors; the first paragraph; page 4. |  |
| Statistical methods | | 12 | (*a*) Describe all statistical methods, including those used to control for confounding | Methods/Statistical Analysis; 2-3 paragraphs; page 5-6. |  |
|  |  |  | (*b*) Describe any methods used to examine subgroups and interactions | Methods/Statistical Analysis; third paragraph; page 5-6. |  |
|  |  |  | (*c*) Explain how missing data were addressed | Table 1 |  |
|  |  |  | (*d*) *Cohort study*—If applicable, explain how loss to follow-up was addressed | N/A |  |
|  |  |  | (*e*) Describe any sensitivity analyses | Methods/Statistical Analysis; fourth paragraph; page 6. |  |
| Results | | | |  |  |
| Participants | 13* | (a) Report numbers of individuals at each stage of study—eg numbers potentially eligible, examined for eligibility, confirmed eligible, included in the study, completing follow-up, and analysed | | Results; first paragraph and figure 1; page 6. | |
|  |  | (b) Give reasons for non-participation at each stage | | Results; first paragraph and table 1; page 6. | |
|  |  | (c) Consider use of a flow diagram | | Figure 1 | |
| Descriptive data | 14* | (a) Give characteristics of study participants (eg demographic, clinical, social) and information on exposures and potential confounders | | Results; table 1 | |
|  |  | (b) Indicate number of participants with missing data for each variable of interest | | Results; table 1 | |
|  |  | (c) *Cohort study*—Summarise follow-up time (eg, average and total amount) | | Methods/Predictive factors; the last paragraph - Page 5. | |
| Outcome data | 15* | *Cohort study*—Report numbers of outcome events or summary measures over time | | Results; third paragraph and table 1; page 8. | |
| Main results | 16 | (*a*) Give unadjusted estimates and, if applicable, confounder-adjusted estimates and their precision (eg, 95% confidence interval). Make clear which confounders were adjusted for and why they were included | | Results; fourth paragraph and table 2; page 8. | |
|  |  | (*b*) Report category boundaries when continuous variables were categorized | | Results; fourth paragraph and table 2; page 8. | |
|  |  | (*c*) If relevant, consider translating estimates of relative risk into absolute risk for a meaningful time period | | N/A | |
| Other analyses | 17 | Report other analyses done—eg analyses of subgroups and interactions, and sensitivity analyses | | Results/Analysis of the subgroup; all paragraphs and table 3 end 4; page 9-10. | |
| Discussion | | | |  | |
| Key results | 18 | Summarise key results with reference to study objectives | | Discussion; | |
| Limitations | 19 | Discuss limitations of the study, taking into account sources of potential bias or imprecision. Discuss both direction and magnitude of any potential bias | | Discussion; fourth paragraph; page 12. | |
| Interpretation | 20 | Give a cautious overall interpretation of results considering objectives, limitations, multiplicity of analyses, results from similar studies, and other relevant evidence | | Discussion; 2-3 paragraphs; page 11-12. | |
| Generalisability | 21 | Discuss the generalisability (external validity) of the study results | | Conclusions; the paragraph; page 13. | |
| Other information | | | |  | |
| Funding | 22 | Give the source of funding and the role of the funders for the present study and, if applicable, for the original study on which the present article is based | | None | |

*Give information separately for cases and controls in case-control studies and, if applicable, for exposed and unexposed groups in cohort and cross-sectional studies.
